# The origin of striation in the metastable β phase of titanium alloys observed by transmission electron microscopy

**DOI:** 10.1107/S1600576717004150

**Published:** 2017-05-08

**Authors:** Jiangkun Fan, Jinshan Li, Yudong Zhang, Hongchao Kou, Jaafar Ghanbaja, Weimin Gan, Lionel Germain, Claude Esling

**Affiliations:** a State Key Laboratory of Solidification Processing, Northwestern Polytechnical University, Xi’an, 710072, People’s Republic of China; b Laboratoire d’Étude des Microstructures et de Mécanique des Matériaux (LEM3), CNRS 7239, Université de Lorraine, Metz, 57045, France; c Laboratory of Excellence on Design of Alloy Metals for low-mAss Structures (DAMAS), Université de Lorraine, Metz, 57045, France; d Institut Jean Lamour, CNRS, Université de Lorraine, Parc de Saurupt, CS50840, Nancy, 54011, France; e German Engineering Materials Science Centre (GEMS) at MLZ, Helmholtz-Zentrum Geesthacht, Garching, D-85748, Germany

**Keywords:** Ti alloys, structure modulation, striation, electron diffraction, transmission electron microscopy, TEM

## Abstract

For the β phase of Ti-5553-type metastable β-Ti alloys, striations in transmission electron microscopy (TEM) bright- and dark-field images have been frequently observed but their origin has not been sufficiently investigated. In the present work, this phenomenon is studied in depth from the macroscopic scale by neutron diffraction to the atomic scale by high-resolution TEM. The results reveal that the β phase contains homogeneously distributed modulated structures, intermediate between that of the β phase (cubic) and that of the α phase or the ω phase (hexagonal), giving rise to the appearance of additional diffraction spots at 1/2, 1/3 and 2/3 β diffraction positions. The intermediate structure between β and α is formed by the atomic displacements on each second {110}_β_ plane in the  direction, whereas that between β and ω is formed by atomic displacements on each second and third {112}_β_ plane in the opposite  direction. Because of these atomic displacements, the {110}_β_ and {112}_β_ planes become faulted, resulting in the streaking of β diffraction spots and the formation of extinction fringes in TEM bright- and dark-field images, the commonly observed striations. The present work reveals the origin of the striations and the intrinsic correlation with the additional electron reflections of the β phase.

## Abstract 2

In the present work, the striations and diffraction characteristics of the β phase of Ti-5553 alloy are thoroughly investigated.

## 1. Introduction   

Metastable β-Ti alloys are important structural materials for aeronautical applications owing to their high strength-to-density ratio, superior strength and ductility combination, and good workability (Raghunathan *et al.*, 2007 ▸; Boyer & Briggs, 2005 ▸; Jackson *et al.*, 2005 ▸). The excellent mechanical properties of these alloys benefit from a microstructure having fine and dispersed α precipitates in the β matrix obtained *via* phase transformation. Because of the presence of a large amount of β-stabilizing (such as Mo, Cr, V, Fe) and α-stabilizing (Al) elements, the structural transformation of Ti involves several transformation stages that produce a variety of metastable phases (β + β′ and ω, α′ or α′′) in addition to the equilibrium α phase. As the type of precipitates and their morphologies, sizes and distributions have a strong influence on the final mechanical properties of the alloys, phase transformation of the oversaturated β phase has been a topic of intensive study (Salib *et al.*, 2013 ▸; Tobe *et al.*, 2014 ▸; Al-Zain *et al.*, 2012 ▸; Lai *et al.*, 2015 ▸; Chen *et al.*, 2014 ▸; Laheurte *et al.*, 2005 ▸).

So far, investigations have focused on the formation conditions and the microstructural features of the stable α and the metastable ω, α′, α′′ and β + β′ phases in Ti alloys. The α phase has a close-packed hexagonal (h.c.p.) structure and can be obtained when the alloy is cooled at low cooling rates from temperatures above the β transus (*T*_β_) or aged in the α precipitation temperature range. The α phase formed possesses certain orientation relationships (ORs) with the parent β phase (Burgers, 1934 ▸; Pitsch & Schrader, 1958 ▸; Potter, 1973 ▸). The diffraction spots of the α phase in transmission electron microscopy (TEM) selected-area electron diffraction (SAED) are located close to the 1/2 β diffraction positions (Jones *et al.*, 2009 ▸). The ω phase has a hexagonal structure and can be obtained either isothermally or athermally. It usually appears as small (nanoscaled) cuboidal or ellipsoidal particles in the β matrix. In general, the athermal ω phase forms during β quenching, whereas the isothermal ω phase forms during ageing at temperatures below 823 K (Sikka *et al.*, 1982 ▸; Ahmed *et al.*, 2015 ▸). It also possesses a specific OR with the β matrix (Furuhara *et al.*, 2001 ▸) and produces diffraction spots at the 1/3{112}_β_ and 2/3{112}_β_ diffraction positions (Ng *et al.*, 2011 ▸; Lai *et al.*, 2015 ▸; Tane *et al.*, 2013 ▸). As for the α′ (hexagonal) and the α′′ (orthorhombic) phases, the two kinds of martensite, they generally form with an acicular or plate shape during rapid cooling from a temperature above or near *T*_β_. Their diffraction spots are located very close to those of the α phase. The solute-depleted β and the solute-enriched β′ (β + β′) phases appear concomitantly with a rod-like morphology and are formed through spinodal decomposition at the early stage of low-temperature ageing. The structures of the two products are cubic (b.c.c.), like the parent β phase, and hence no diffraction spots other than those of β appear in the SAED patterns (Lütjering & Williams, 2007 ▸).

Structure instability of the β phase has also been a topic attracting attention (Nag *et al.*, 2011 ▸; Bennett *et al.*, 2015 ▸; Zheng *et al.*, 2016 ▸; Abdel-Hady *et al.*, 2007 ▸; Ng *et al.*, 2011 ▸; Barriobero-Vila *et al.*, 2015 ▸; Li *et al.*, 2016 ▸; Williams *et al.*, 1971 ▸; Sukedai *et al.*, 1997 ▸). Atomic scaled lattice modulations have been revealed in some binary and ternary Ti–Nb (Tahara *et al.*, 2011 ▸; Zheng *et al.*, 2016 ▸) and Ti–Mo (Nag *et al.*, 2011 ▸; Devaraj *et al.*, 2012 ▸) alloys through examining the diffuse streaks in TEM SAED patterns and the atomic correspondences in high-resolution TEM. Two kinds of lattice modulations ({110}_β_ 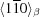 and {112}_β_ 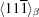) have been shown to give rise to the formation of additional diffraction spots between the fundamental diffraction spots of β. The origins of these lattice modulations and their impact on phase transformation of the β phase in the corresponding alloys have been intensively studied, and knowledge of the structure instability of Ti alloys and their phase transformation characteristics has been advanced. Although the structure modulations of the β phase at the atomic scale have been clarified, striations in the microscopic scale frequently observed in TEM bright- and dark-field images – another phenomenon of the β phase – have not been addressed sufficiently. These striations have been interpreted either as stacking faults (Huang *et al.*, 2011 ▸) or as spinodal decomposition zones (Barriobero-Vila *et al.*, 2015 ▸). However, such interpretations have not been experimentally confirmed. Moreover, whether these microscopic striations are linked with the atomic structure modulations is not known.

Motivated by such observations, we performed a thorough investigation on Ti–5Al–5Mo–5V–3Cr (Ti-5553), a representative alloy of the metastable β-Ti alloys, with the aim of elucidating the above questions.

## 2. Material and experimental details   

The Ti-5553 alloy used in the present work was multi-directionally forged in the α + β temperature range. The chemical composition measured by a spectrograph is Al: 5.26; Mo: 4.99; V: 4.80; Cr: 2.86; Fe: 0.42 in wt%. The β transus temperature (*T*_β_) determined by metallographical analyses is about 1143 K.

Cylindrical specimens, 10 mm in diameter and 15 mm in height, were machined out of the forged bar and solution treated at 1173 K for 30 min in order to obtain a microstructure of single β phase. After solution treatment, some specimens were further aged at 1073 and 873 K, to allow part of the β phase to transform to α phase. The heat-treatment parameters are given in Table 1 ▸. The specimens were loaded into the furnace when the set temperature was reached.

Neutron diffraction measurements were performed at room temperature, using the texture and stress diffractometer STRESS-SPEC at Heinz Maier-Leibnitz Zentrum (MLZ), Garching, Germany, to determine the phase constituents and the lattice parameters of the β phase in the solution-treated specimen. Monochromatic neutrons (0.1689 nm) were obtained using a Ge(311) monochromator. Diffraction patterns in the 2θ range from 35 to 100° were acquired. The incident-beam size was Ø 15 mm, ensuring that the specimen of Ø 10 × 15 mm was entirely bathed in the beam. During the measurement, the cylindrical specimen was continuously rotated around its axis. A standard Si powder was measured to obtain the instrumental parameters.

Mesoscaled microstructures of the as-forged and heat-treated specimens were examined by scanning electron microscopy (SEM), using a JEOL JSM-6500F field emission gun scanning electron microscope. Microstructural observation specimens were prepared with mechanical grinding and then chemical etching, using a modified Kroll’s reagent of 10 vol.% HF, 10 vol.% HNO_3_ and 80 vol.% H_2_O, for 10 s.

Nanoscaled and atomic scaled microstructures were analysed by TEM, using a Philips CM 200 transmission electron microscope, a JEOL JEM-ARM200F high-resolution transmission electron microscope and an FEI Tecnai G2 F30 high-resolution transmission electron microscope. High-angle annular dark-field (HAADF) images were acquired with an inner and outer collecting angle of 50 and 180 mrad, respectively. Chemical compositions were analysed by TEM energy-dispersive X-ray spectrometry (EDS) in line scanning mode. TEM thin films were prepared by first mechanical thinning to 100 µm and then electrolytic polishing to perforation at 238 K in a solution of 20% perchloric acid in methanol at a voltage of 10 V, using a Struers Tenupol-5 twin-jet electropolisher. The crystallographic orientations of the microstructural constituents expressed in a triplet of Euler angles in Bunge’s notation (Bunge *et al.*, 1981 ▸) were determined by indexing the TEM Kikuchi line patterns, using an in-house designed software package, *Euclid Phantasies* (*EP*) (Morawiec, 1999 ▸; Morawiec *et al.*, 2002 ▸). The observed traces of the striations were identified with a trace analysis method (Zhang *et al.*, 2011 ▸). Firstly, the theoretical trace orientations of all low-indexed crystalline planes of the corresponding phase in the TEM screen reference system were calculated using the determined crystallographic orientation of the phase. Secondly, the calculated traces were compared with the observed ones. Finally, the plane having the best matched trace was determined as the plane giving rise to the striations. To avoid an accidental match, at least three specimen tilts were utilized to verify each identified plane. The analysis of atomic correspondences and the simulation of single-crystal electron diffraction were performed using the *CrystalMaker* software package and *EP* (Morawiec, 1999 ▸; Morawiec *et al.*, 2002 ▸).

## 3. Results and discussion   

### 3.1. Microstructural characteristics   

**Figure 1:**
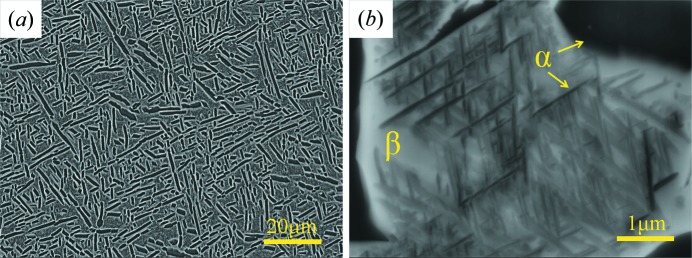
SEM backscattered electron micrographs of the as-forged microstructure of Ti-5553 alloy. There are two sizes of α phase: (*a*) large globular and rod like and (*b*) fine needle-shaped α between coarse α.

The microstructure of the present (α + β) forged Ti-5553 is characterized by the typical constituents of Ti-5553 alloys, consisting of coarse α lamellas, as shown in Fig. 1 ▸(*a*). Between the coarse α, there are fine α lamellas, as shown in the magnified SEM micrograph in Fig. 1 ▸(*b*). Both are distributed homogeneously in the β matrix.

**Figure 2:**
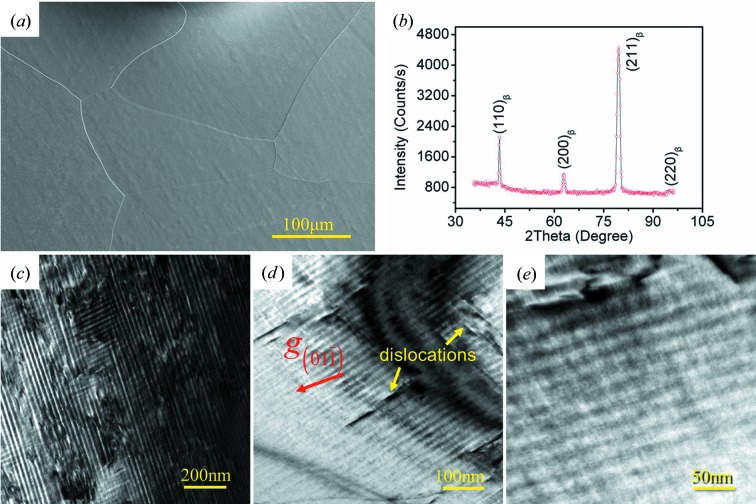
(*a*) SEM secondary electron (SE) micrograph of Ti-5553 alloy after solution treatment (holding at 1173 K for 30 min and quenching in water), showing the single β phase having equiaxed grains. (*b*) Neutron diffraction pattern. (*c*) TEM bright-field micrograph showing the striated structure in β grains. (*d*) TEM bright-field micrograph showing the improved contrast of the striations across the 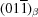 extinction contour (the thick dark lines), where the deviation of the 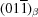 reflecting plane from the Bragg position, *s_g_*, is zero and the two-beam condition is approximately met. The reciprocal vector 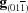 is indicated. (*e*) Magnified TEM bright-field micrograph of an area in (*d*), showing fine strips within the striations.

After the specimen has been solution treated, all α constituents dissolve into the β matrix, as shown in Fig. 2 ▸(*a*). Only equiaxed β grains with an average grain size of ∼350 µm are present. This phase constituent is confirmed by the in-volume neutron diffraction analysis. The diffraction pattern is shown in Fig. 2 ▸(*b*). Only the β phase having a b.c.c. structure with lattice constant *a* = 3.2355 Å is identified. However, a close examination by TEM reveals that a large number of striations with two grey levels are distributed alternately within the β matrix, as shown in Fig. 2 ▸(*c*). The inter-striation spacing (the width of one pair of dark and light bands) is about several tens of nanometres. The length direction of the striations is not fixed but dependent on the orientation of the crystal. The striations are oriented in more than one direction, forming a tweed pattern. The contrast is particularly high in the vicinity of extinction contours, for example the 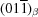 extinction contour in Fig. 2 ▸(*d*). In fact, within the extinction contours, the two-beam condition and the zero deviation of the reflecting plane from the Bragg position (*s_g_* = 0) are approached. Although the contrast of the striations can be distinguished, the edges of each striation are not sharply defined, unlike the case of dislocations or stacking faults where the contrast change is sharp (Lu *et al.*, 2009 ▸). Moreover, within the striations, the contrast is not constant, as shown in Fig. 2 ▸(*e*), the magnified image of an area in Fig. 2 ▸(*d*). Fine strips are found to run in two directions. The fact that these fine structures are visible in greater contrast in the area very close to the extinction contour where *s_g_* = 0 and the two-beam condition are approximately satisfied indicates that these contrasts are of crystallographic origin. Dislocations with higher contrast can also be seen in the striated regions, as indicated in Fig. 2 ▸(*d*). Comparing Fig. 2 ▸ with Fig. 1 ▸, we can see that the striated structure does not bear any morphological or distributional resemblances to the two scaled α phases in the as-forged microstructure. This fact rules out the possibility that the striations are the residual α regions that did not fully transform to β during solution treatment.

**Figure 3:**
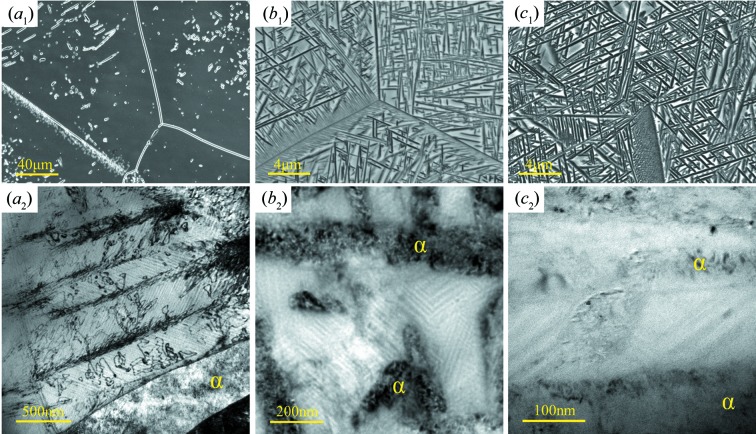
(*a*_1_) SEM SE and (*a*_2_) TEM micrographs of Ti-5553 alloy aged at 1073 K for 30 min. (*b*_1_) SEM SE and (*b*_2_) TEM micrographs of Ti-5553 alloy aged at 873 K for 30 min. (*c*_1_) SEM SE and (*c*_2_) TEM micrograph of Ti-5553 alloy aged at 873 K for 180 min.

Considering that the β phase is retained by fast cooling during solution treatment, the oversaturation of α-stabilizing elements in β, like Al, may have some influence on the formation of the striated microstructure. To verify this possibility, the aged specimens (aged at 1073 and 873 K) were examined. The microstructures obtained using SEM and TEM are shown in Fig. 3 ▸. It is seen that, with decreasing ageing temperature, the amount of α phase increases. The precipitation sites and morphologies of the α phase for the two ageing conditions are also typical of those of Ti-5553 alloys (Phelan *et al.*, 2006 ▸; Flower, 1990 ▸). When the ageing temperature is 1073 K (Fig. 3 ▸ *a*_1_), the α phase forms mainly at β grain boundaries and occasionally in the grain interiors. When the ageing temperature is 873 K for 30 min, a large number of plate-shaped α precipitates form in the interior of the β grains and the Widmanstätten α precipitates in the regions along certain β grain boundaries (Fig. 3 ▸ *b*_1_). When the ageing time is prolonged to 180 min, the amount of α phase does not increase too much (Fig. 3 ▸ *c*_1_), showing a tendency of saturation. However, TEM examination reveals that, besides the formation of a larger number of dislocations, the remaining β phase still contains striations as seen in Figs. 3 ▸(*a*_2_), 3 ▸(*b*_2_) and 3 ▸(*c*_2_), as in the case of the solution-treated β phase. This indicates that the striations are not related to the oversaturation of the α-stabilizing elements. Moreover, the α phase formed does not show any morphology of parallel lamellas.

### 3.2. Chemical composition distribution characteristics   

**Figure 4:**
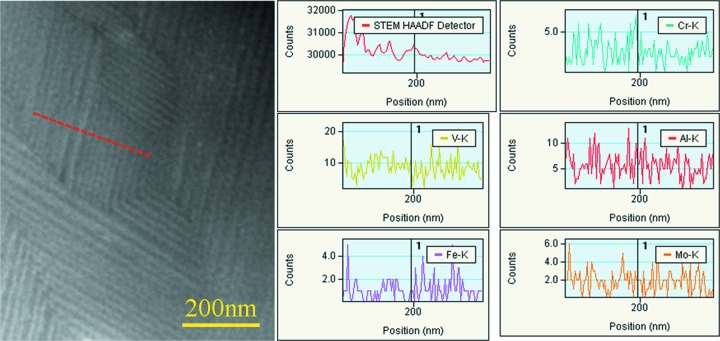
STEM HAADF image of the striated structure in the solution-treated specimen and the EDS line profiles of Cr, V, Al, Fe and Mo measured along the line indicated in the STEM HAADF image.

To verify if the striations are related to composition oscillation of the alloying elements in the β phase, STEM-HAADF-EDS measurements (STEM is scanning transmission electron microscopy) were performed on the solution-treated specimen. The line profiles of the distribution of Ti, Al, V, Cr, Mo and Fe across the striations do not display any repartition of these elements from one striation to another. Fig. 4 ▸ shows one example of the measurements. This result indicates that behind the contrast change from one striation to another there is no alloying element repartition.

### 3.3. Identification of substructures in β phase   

**Figure 5:**
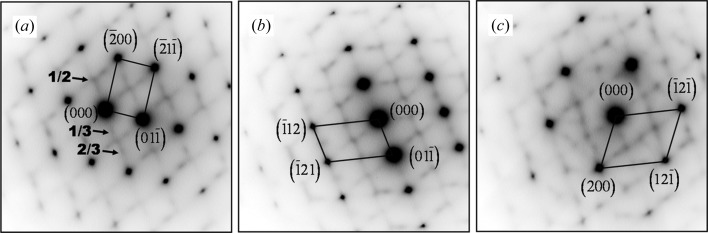
TEM SAED patterns of the striated structure. (*a*) [011]_β_, (*b*) [113]_β_ and (*c*) [012]_β_ zone axis patterns.

In order to further explore the possible origins of the striations, several β zone axis TEM SAED patterns, namely 〈100〉_β_, 〈011〉_β_, 〈111〉_β_, 〈113〉_β_, 〈133〉_β_ and 〈012〉_β_, were acquired and analysed. The typical [011]_β_, [113]_β_ and [012]_β_ patterns frequently used in the literature are given in Fig. 5 ▸. It is seen that in each pattern there are three sets of diffraction spots. Among these sets, one is of high-intensity spots from the b.c.c. β and the other two are of low intensities located at 1/2 β diffraction positions, and at 1/3 and 2/3 β diffraction positions. The low-intensity spots at the 1/2 β diffraction positions are close to the diffraction positions of the α phase but not exactly the same (hereafter we denote this diffraction as quasi-α diffraction), whereas the spots at 1/3 and 2/3 β diffraction positions are very close to those of the ω phase (hereafter we denote this diffraction as quasi-ω diffraction). The two types of diffraction possess one common feature: the presence of fine streaks continuous between the diffraction spots of the β phase and passing through the origin (transmitted beam) of the diffraction pattern. This is the characteristic of the scattering effect arising from crystal size (Tanner, 1966 ▸). These fine streaks indicate that the domains producing the corresponding 1/2, 1/3 and 2/3 β diffraction are very small. As the sizes of the domains are too small, no visible dark-field contrast can be obtained using the diffracted beams at the 1/2, 1/3 and 2/3 β diffraction positions. The same situation was also encountered in another study (Inaekyan *et al.*, 2015 ▸). According to the analyses made by Tahara, Zheng, Devaraj and co-workers, using Ti–Nb-based and Ti–Mo alloys (Tahara *et al.*, 2011 ▸; Zheng *et al.*, 2016 ▸; Devaraj *et al.*, 2012 ▸; Nag *et al.*, 2011 ▸), the additional diffraction spots are produced by specific {110}_β_ 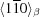 and {112}_β_ 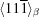 lattice modulations. To verify such situations in the present Ti-5553 alloy, high-resolution TEM (HRTEM) observations were performed.

**Figure 6:**
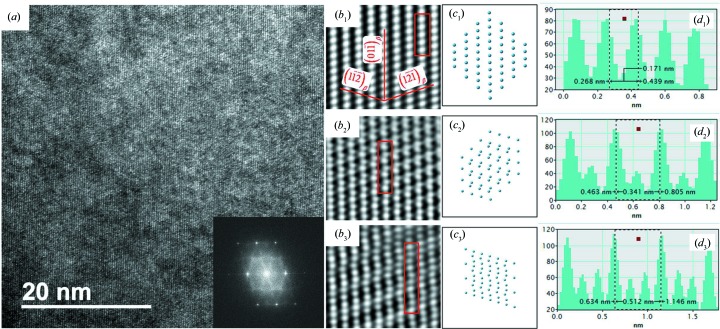
(*a*) HRTEM micrograph of the striated structure viewed in the [311]_β_ direction. The inset shows an FFT image from the corresponding HRTEM image. (*b*) Characteristic zones corresponding to the perfect β phase (*b*_1_), those giving quasi-α diffraction (*b*_2_) and those giving quasi-ω diffraction (*b*_3_) produced by the inverse FFT using the Bragg reflections. (*c*) Projected atom columns from the corresponding constructed structures, including perfect β (*c*_1_), two-layer modulated (*c*_2_) and three-layer modulated (*c*_3_) structures, and (*d*) intensity profiles of the outlined (in red) atom columns in (*b*), including perfect β (*d*_1_), two-layer modulated (*d*_2_) and three-layer modulated (*d*_3_) structures.

Fig. 6 ▸(*a*) shows an HRTEM micrograph acquired in the area with one of the 〈311〉_β_ directions parallel to the incident-beam direction. The inset is the corresponding diffraction pattern generated by the fast Fourier transformation (FFT) from the HRTEM image. The pattern reproduces well the characteristics of the corresponding SAED 〈311〉_β_ zone axis pattern (Fig. 5 ▸ *b*). The image generated by the inverse FFT using the Bragg reflections in the FFT diffraction pattern clearly shows the morphology and the atomic correspondences of the structures giving the β diffraction, the quasi-α diffraction (1/2 β diffraction positions) and the quasi-ω diffraction (1/3 and 2/3 β diffraction positions). The inverse FFT images (Fig. 6 ▸ *b*) and the projected atom columns (Fig. 6 ▸ *c*) from the corresponding constructed structures together with the intensity profiles (Fig. 6 ▸ *d*) of the atom columns constituting the 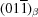 plane are also provided. It is seen from the inverse FFT image and the intensity profile in Figs. 6 ▸(*b*_1_) and 6 ▸(*d*_1_) that, for the ideal β phase, the atoms constituting the 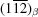 planes [or 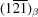 planes] with an interplanar spacing of 1.35 Å and 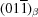 planes with an interplanar spacing of 2.33 Å are in the positions to allow constructive interference of the diffracted electron waves (in phase position). The {112} layers of the b.c.c. structure are clearly visualized. These can be used as references to differentiate the structures giving the quasi-α diffraction and the quasi-ω diffraction. As seen from Fig. 6 ▸(*b*_2_), for the structure producing quasi-α diffraction, every first and third 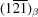 plane in the 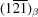 stacks is clearly in the b.c.c. position with an interplanar spacing of 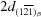, whereas the atoms on every second 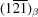 plane are obviously displaced as seen in the outlined zone. The intensities of the displaced atoms in the intensity profile in Fig. 6 ▸(*d*_2_) are much lower with respect to those of undisplaced ones, indicating that these atoms are out of positions, allowing constructive interference of the diffracted waves (out of phase position). Such atomic displacements transform the ideal b.c.c. (β) structure to a two-layered modulated structure and make the additional diffraction spots appear at 1/2 {112}_β_ diffraction positions. This situation is similar to the cases found in Ti–23Nb–1.0O by Tahara *et al.* (2011 ▸) and in Ti–26Nb–2Zr by Zheng *et al.* (2016 ▸), even though the alloying elements in the present alloy are totally different from those in the two above-mentioned alloys. For the structure producing quasi-ω diffraction, the displacements happen in two consecutive 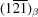 layers in opposite directions, and the atoms in every first and fourth 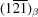 layer stay in the exact positions of the β b.c.c. structure, as seen in the outlined zone in Fig. 6 ▸(*b*_3_). The intensity profile also implies that the displaced atoms are no longer in phase positions (Fig. 6 ▸ *d*_3_). Such a structural change forms a three-layered modulation. Owing to the structure modulation in the two consecutive 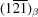 planes, additional diffraction spots at the 1/3 and 2/3 β diffraction positions appear. This situation is similar to that found by Nag and co-workers (Nag *et al.*, 2011 ▸; Devaraj *et al.*, 2012 ▸) in a binary Ti–Mo alloy. According to their work, the formation of such a structure modulation is realized by displacive collapse of the 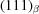 planes of the parent b.c.c. structure, forming ω-like embryos (Nag *et al.*, 2011 ▸; Devaraj *et al.*, 2012 ▸).

**Figure 7:**
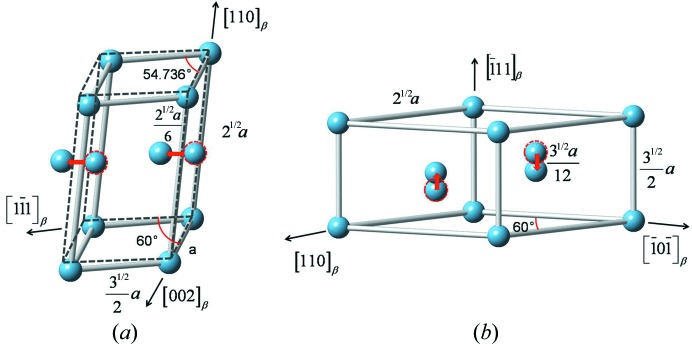
Structural model of (*a*) two-layered modulation and (*b*) three-layered modulation with respect to the ideal cubic β structure. For comparison, the ideal α is outlined in dashed lines.

**Figure 8:**
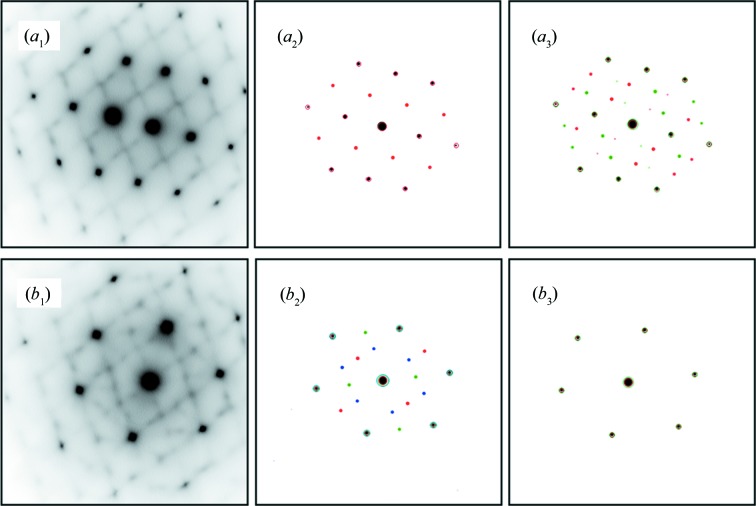
Experimental and simulated diffraction patterns: (*a*_1_), (*b*_1_) experimental [011]_β_ and [012] zone axis diffraction patterns, (*a*_2_), (*b*_2_) simulated diffraction patterns of the two-layered modulation from six different variants. (*a*_3_), (*b*_3_) simulated diffraction patterns of the three-layered modulation from four different variants. The reflections from different variants are shown by different colours.

The atomic correspondences revealed by the HRTEM analyses demonstrate that the two-layered modulated structure and the three-layered modulated structure possess structural resemblance to both the cubic structure of the β phase (for the undisplaced atoms) and the hexagonal structure of the α phase or the ω phase (for the displaced atoms). On the basis of such characteristics and measured displacement amounts for the two structure modulations, we constructed the two modulated structures, following the Burgers OR between α and β (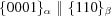; 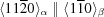) (Burgers, 1934 ▸) and the OR between ω and β (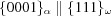; 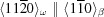) (Furuhara *et al.*, 2001 ▸), as shown in Fig. 7 ▸. The unit cell of the structure giving quasi-α diffraction (Fig. 7 ▸ *a*) is encased in one pair of {110}_β_ (basal plane) planes, one pair of {112}_β_ planes and one pair of {101} planes (prismatic planes). The only difference from the cubic structure of the β phase is that the atoms on {110}_β_ between the basal planes are displaced in the 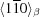 direction by an amount *a*/6 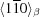, as indicated in Fig. 7 ▸(*a*). For comparison, the ideal α structure is plotted as dashed lines in the figure. Using this structure model, the diffraction patterns of all acquired zone axes were simulated, using the software package *CrystalMaker*. With six possible variants (there are six distinct {110}_β_ planes), all the observed diffraction spots corresponding to the quasi-α diffraction are reproduced and properly indexed. As examples, the experimental and simulated 〈011〉 and 〈012〉 β zone axis diffraction patterns are shown in Figs. 8 ▸(*a*_1_), 8 ▸(*a*_2_) and 8 ▸(*b*_1_), 8 ▸(*b*_2_), where spots from different variants of the {110}_β_/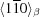 displacement are in different colours. The [311]_β_ (the 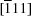 for the modulated structure produced by the {110}_β_/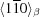 displacements) projection of the atom columns is given in Fig. 6 ▸(*b*_2_). The good match of the constructed structure in both direct space (Fig. 6 ▸ *b*_2_) and reciprocal space (Figs. 8 ▸ *a*_2_ and 8 ▸ *b*_2_) validates the structure model and confirms the existence of the *a*/6 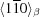 atomic displacements on the six distinct {110}_β_ planes. This structure is in general equivalent to the O′ structure found by Zheng *et al.* (2016 ▸) in a Ti–Nb–Zr alloy, but the amount of displacement or shuffle is further detailed (*a*/6 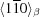) in the present alloy.

The unit cell of the modulated structure produced by the {121}_β_/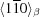 displacements shown in Fig. 7 ▸(*b*) is encased in one pair of 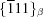 (basal planes) and two pairs of {112}_β_ planes (prismatic planes). The difference from the structure of the ideal β phase is that the atoms on the two 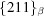 (or 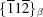) planes between the prismatic planes are displaced in opposite 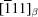 directions with a distance between 0 and *a*/12 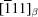, as indicated in Fig. 7 ▸(*b*). It should be noted that, if the dis­placement is exactly *a*/12 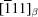, the 1/3 or 2/3 reflections will disappear in some zone axis patterns and this has not been found in the present work. As in the case of the modulated structure produced by the {110}_β_/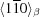 displacements, all the diffractions in the six zone axis patterns are reproduced and properly indexed using four variants, as there are four distinct {111}_β_ planes. Figs. 8 ▸(*a*_3_) and 8 ▸(*b*_3_) show the simulated 〈011〉_β_ and 〈012〉_β_ zone axis patterns. The [311]_β_ (the 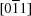 for the modulated structure produced by the {121}_β_/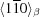 dis­place­ments) projection of the atom columns is given in Fig. 6 ▸(*b*_3_). The good match of the modelled structure in both direct (Fig. 6 ▸ *b*_3_) and reciprocal space (Figs. 8 ▸ *a*_3_ and 8 ▸ *b*_3_) validates the structure model and confirms the existence of the approximate *a*/12 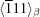 displacements on the 12 distinct {112}_β_ planes. In fact, the atomic displacements on the {112}_β_ planes in the opposite 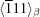 directions realize the ‘collapse’ of the 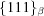 planes, a term used by Nag and co-workers (Nag *et al.*, 2011 ▸; Devaraj *et al.*, 2012 ▸), to form the ω-like embryos (Nag *et al.*, 2011 ▸; Devaraj *et al.*, 2012 ▸). In the present Ti-5553 alloy, the amount of the collapse is less than 0.5*d*_222_. It is not fixed in each domain but diminishes progressively when approaching the outer area of the domain, as shown in Fig. 6 ▸(*b*_3_). Such a state corresponds to the partial collapse of the planes (Nag *et al.*, 2011 ▸; Devaraj *et al.*, 2012 ▸).

The HRTEM examinations reveal that the two modulated structures are in equiaxed shape. Each domain contains about several tens of atoms. They are distributed homogeneously within the β matrix. Clearly, they do not organize in the form of the striations as seen in lower-magnification TEM micrographs (Fig. 2 ▸ *c*). The striations should have some other origins.

### 3.4. Relation between lattice modulation and striations   

**Figure 9:**
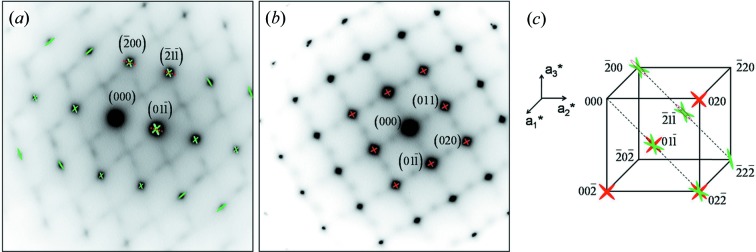
Diffraction patterns of (*a*) [011]_β_ and (*b*) [100]_β_ zone axes. The 〈110〉 relrods are marked in red and the 〈112〉 relrods are marked in green. (*c*) Geometrical relation between the [100]_β_ and the [011]_β_ zone axis patterns in reciprocal space.

Through systematic examination of the SAED patterns acquired in the present work, we found two characteristics associated with the diffraction spots of the β phase. First, some β diffraction spots are obviously elongated in certain reciprocal directions, forming the so-called relrods (Tanner, 1966 ▸; Robertson & Wayman, 1983 ▸). The elongation increases with increasing order of the diffraction. This indicates that the elongation is rather from the scattering effect by crystal defects than by crystal size. Second, the relrods exhibit characteristic extinction. This further confirms that the formation of the relrods is indeed from crystal defects. In the present alloy, the β diffraction spots are elongated in two families of reciprocal directions: 〈110〉 and 〈112〉 (hereafter 〈110〉 relrods and 〈112〉 relrods), as shown in Fig. 9 ▸. In the figures, the 〈110〉 relrods are displayed in red and the 〈112〉 relrods in green, and the geometrical relation between the two zone axes in the reciprocal space with the presence of the two kinds of relrods is also displayed. It is seen from Fig. 9 ▸ that the 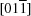 relrods (illustrated by the dashed red line) are absent in the [011]_β_ zone axis pattern (Fig. 9 ▸ *a*) but present in the [100]_β_ zone axis pattern (Fig. 9 ▸ *b*). It is well established that the atomic distortion on certain planes will result in the extension of the diffracted intensity in the direction normal to these planes (Tanner, 1966 ▸; Robertson & Wayman, 1983 ▸). In the present case, the planes normal to the 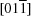 relrods are the 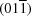
_β_ planes, as the structure is cubic. As analysed above, the formation of the two-layer modulated structure involves atomic displacements on {110}_β_ planes and in 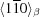 directions. The appearance of the 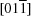 relrods could be related to the formation of this structure. If we denote the displacement vector by **u**, the absence of the 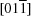 relrods in the [011]_β_ zone axis pattern corresponds to the situation **g** · **u** = 0, where **g** is the reciprocal vector of the diffraction spot. However, in the [100]_β_ zone axis pattern, this situation is not fulfilled, as summarized in Table 2 ▸. This indicates that the formation of the 〈110〉 relrods does indeed originate from the atomic displacements on the 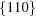
_β_ planes in the 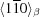 direction, forming the two-layer modulated structure. Such a phenomenon has also been shown in a Ti–Nb–O alloy (Tahara *et al.*, 2011 ▸). Similarly, the formation of the 〈112〉 relrods originates from the atomic displacements forming the three-layer modulated structure.

For a defected crystal, when the atoms are displaced from their lattice positions by a vector **u**, a phase factor α = 2π**g** · **u** is introduced into the diffracted beam **g**. Thus, in the bright-field image formed by excluding the diffracted wave **g** with an objective aperture, we obtain some dark regions arising from the enhanced diffraction by the strain field of the crystal defect (Edington, 1975 ▸). The above extinction of the 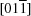 relrods in the [011] zone axis pattern was thus checked with the microstructure. The specimen was tilted to several positions. No specific contrast or striations appeared in the direction perpendicular to 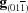, demonstrating the **g** · **u** = 0 invisible character. This character indicates that the contrast of the striations is related to crystal defects. The oscillation of the contrast in the microstructure suggests that the crystal defects possess a planar feature, like stacking faults. Thus, the periodic contrast present on the exit site of the specimen is simply a representation of periodic extinction of the related distorted planes through the thickness of the specimen. With the known geometrical relations of the normal of the distorted planes, the normal of the specimen surface and the direction of the incident beam, the extinction distance of the distorted planes ξ_*g*_ = πΩcosθ_*hkl*_/[λ*f*(θ_*hkl*_)] (Edington, 1975 ▸) can be calculated. In the equation, Ω is the volume of the unit cell, θ_*hkl*_ the Bragg angle, λ the electron wavelength and *f*(θ*_hkl_*) the atomic scattering amplitude (Edington, 1975 ▸). Then, the inter-striation spacing can be derived.

**Figure 10:**
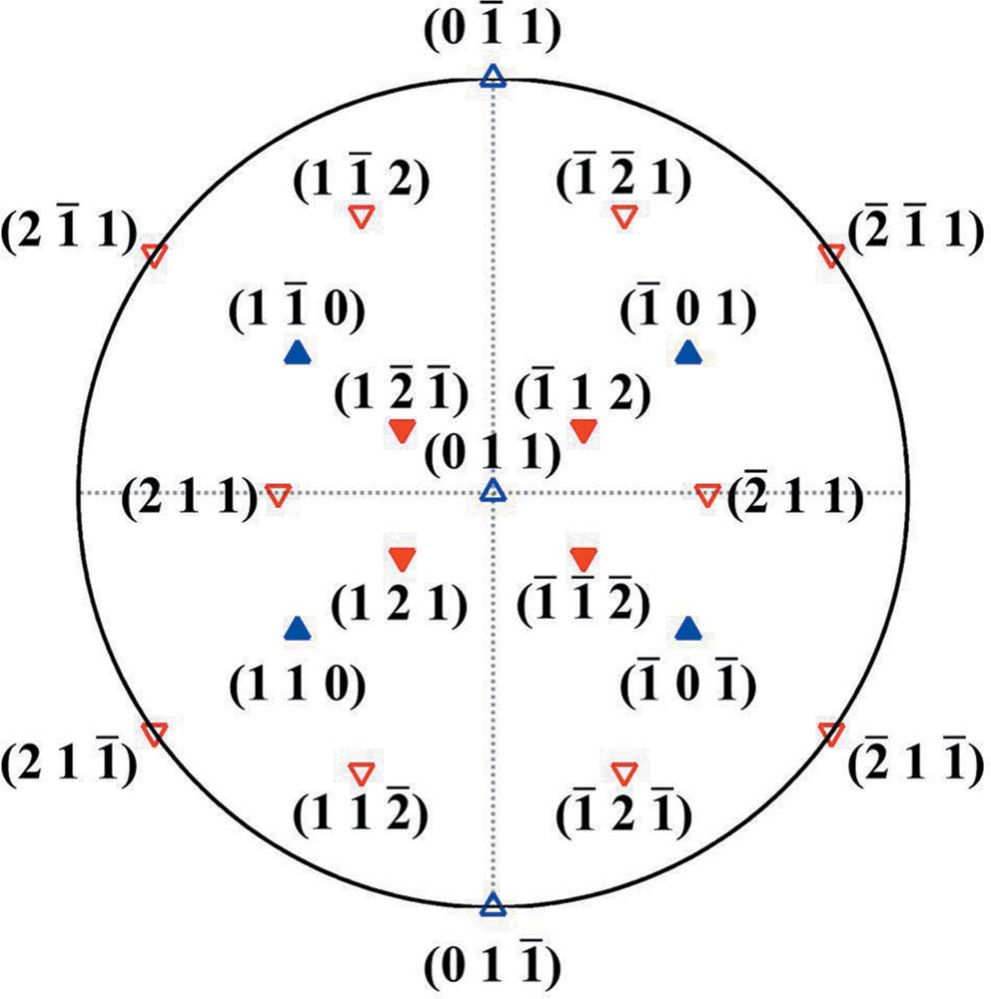
Stereographic projections of the {110}_β_ planes (in blue) and {112}_β_ planes (in red) on the (011)_β_ plane. The visible planes using the 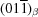 reflection are expressed as solid symbols.

**Figure 11:**
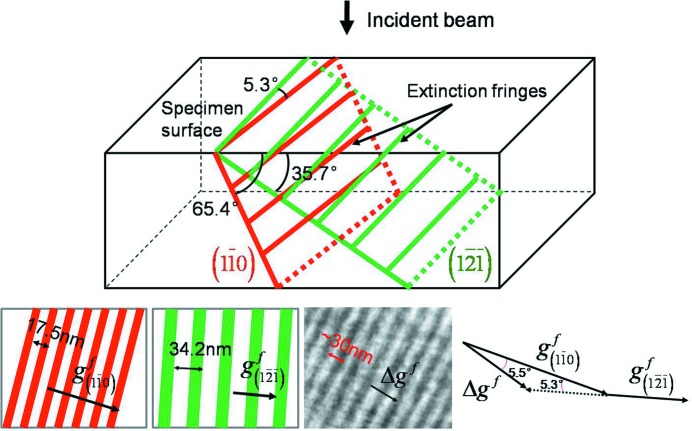
Geometrical positions of the visible 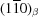 and 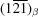 planes in the foil with respect to the incident electron beam and the calculated inter-striation spacing and orientation of the two visible planes. In the figure, 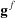 is the reciprocal vector of a given set of fringes, *i.e.* it is perpendicular to the fringe direction and its modulus is the inverse of the inter-fringe spacing.

**Figure 12:**
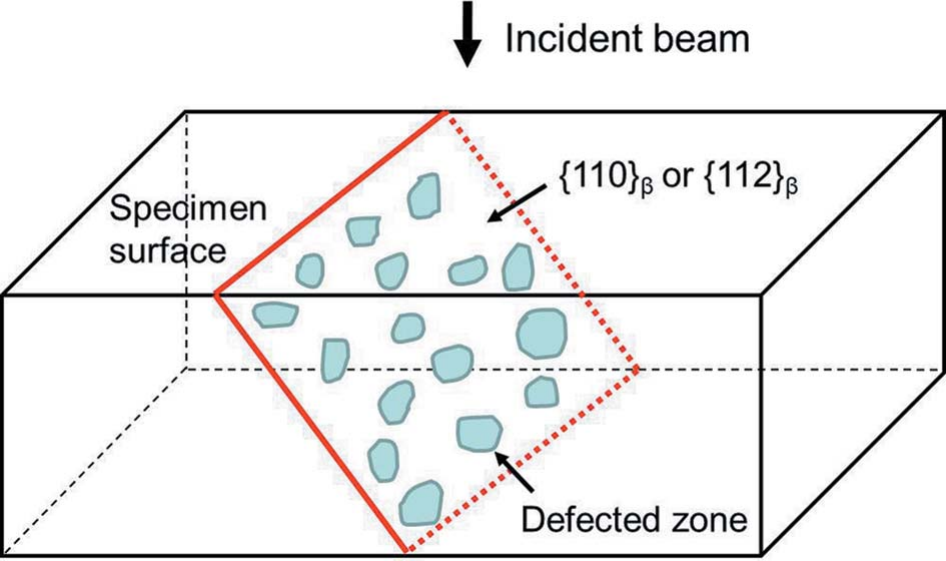
Illustration of nanoscaled atomic displacements on 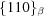 or 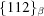 planes that produce microscopic fringes under TEM diffraction contrast.

To simplify the analysis, we used a zone where the specimen surface is very close to the (011)_β_ plane of the β phase [7.76° away from (011)_β_]. The microstructure is displayed in Figs. 2 ▸(*d*) and 2 ▸(*e*). 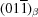 is used as the operative reflecting plane. The spacing of the striations at the 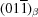 extinction contour (where the two-beam condition and the *s_g_* = 0 condition are approximately satisfied) was measured. It is about 30 nm. Under the two conditions, the formula of the extinction distance ξ*_g_* has a particularly simple form as given above. The visibility of the defected {110}_β_ and {112}_β_ planes under the 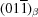 reflection is verified and the results are given in Table 3 ▸. The orientations of these planes with respect to the specimen surface are represented with the stereographic projection on the (011)_β_ plane (the approximate specimen surface) and displayed in Fig. 10 ▸, where the visible planes are plotted as solid symbols. It is seen that two pairs of {110}_β_ planes [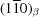 and 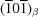, 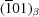 and 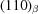] and two pairs of {112}_β_ planes [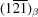 and 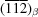, 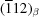 and 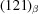] are visible. The poles of 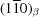 and 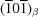 and 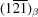 and 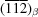 are located on the same diameter of the stereographic projection, as seen in Fig. 10 ▸, and thus these planes share approximately the same orientation of the intersection with the specimen surface, as the surface is very close to the (011)_β_ plane. The situation is similar for 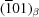 and 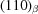 and 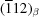 and 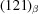. The geometrical relations of the 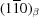 and the 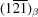 plane with respect to the incident beam are illustrated in Fig. 11 ▸. The spacing of the extinction fringes of the two planes on the exit site of the specimen is calculated. It is 17.5 nm for the 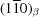 plane and 34.2 nm for the 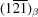 plane. As the two sets of fringes are both projected on the TEM screen, the eventually observed fringes should be a mixture of the two and each can be treated as a set of Moiré fringes, as illustrated in Fig. 11 ▸. Thus, the spacing and the orientation of the mixed fringes can be calculated according to the equations given by Edington (1975 ▸). The calculated spacing of the mixed fringes is 35.5 nm and the angle between the mixed fringes and the 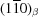 fringes is 5.5°. The results are indicated in Fig. 11 ▸. This spacing is very close to the observed inter-striation spacing and the orientation of the calculated fringes is also very close to that of the observed striations. This confirms that the striations do indeed originate from the atomic displacements on the {110}_β_ and {112}_β_ planes, forming the modulated structures. The diffuse contrasts within each striation indicate that the atomic displacement is not constant on the entire defected plane, as in the ideal case of stacking faults, but localized in the atomic scale or nanoscale and homogeneously dispersed on the plane, as illustrated in Fig. 12 ▸. This confirms that the striations do indeed originate from the two kinds of structure modulations. The fact that only one type of the oriented striations is clearly observed in each striation zone indicates that the atomic displacements on the other potentially visible planes are not prevalent and the striations in these directions are less visible. They act as background noise to further perturb the contrast of the prevalent striations. However, in different zones, the other oriented striations are prevalent, as shown in Fig. 2 ▸(*c*). From the diffraction patterns given in Fig. 5 ▸ that were taken in relatively large sample areas, one can see that all the variants of the two families of displacements exist but their direction visibility under TEM diffraction contrast is conditional. Hereby the origin of the striation of the β phase is elucidated.

## 4. Summary   

In the present work, the striations and the diffraction characteristics of the β phase of Ti-5553 alloy were thoroughly investigated. The results demonstrate that the structure of the β phase is not ‘purely’ body-centred cubic. A large amount of structure modulation at the atomic scale exists in the β phase, with two distinct structures intermediate between that of the β phase and that of the α or the ω phase. These structures are formed by atomic displacements on each second {110}_β_ plane in the 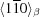 direction and by atomic displacements on each second and third {112}_β_ plane in opposite 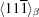 directions, giving rise to the additional diffraction spots at 1/2, and 1/3 and 2/3 β reflection positions. Because of these atomic displacements, the {110}_β_ and {112}_β_ planes become distorted, resulting in the streaking of the β diffraction spots and the formation of extinction fringes in the TEM bright- and dark-field images, the striations. Different from the diffraction contrast induced by perfect stacking faults, the contrast of the striations is diffuse and not constant, as the distorted regions on the corresponding planes are very small at the atomic scale. In the microscopic scale, the striations are distributed homogeneously, implying that the formation of the new structures is global and homogeneous. The results of the present work provide new information on the origins of the striations revealed under TEM diffraction contrast and their intrinsic link with specific electron diffraction phenomena.

## Figures and Tables

**Table 1:** Heat-treatment parameters of the Ti-5553 alloy

Solution treatment			Ageing treatment		
Temperature (K)	Time (min)	Cooling	Temperature (K)	Time (min)	Cooling
1173	30	Water quenching	873	30/180	Water quenching
			1073	30	

**Table 2:** **g** · **u** for the [011]_β_ and [100]_β_ zone axis diffraction

Zone axis	[011]_β_			[100]_β_		
**g**	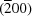	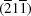	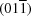	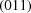	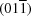	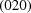
**u** = [011] on the distorted plane 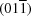	0	0	0	≠0	≠0	≠0

**Table 3:** Visibility of the distorted {110}_β_ and {112}_β_ planes under 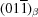 reflection

Distorted plane	{110}_β_						{112}_β_			
							±(112)	± 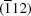	± 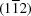	± 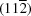
	± 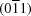	± 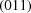 †	± 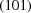	± 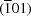	± 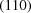	± 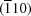	± 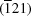	±(121)	± 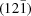	± 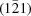
							± 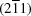 †	± 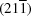 †	±(211)	± 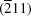
**u**	±[011]	± 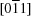	± 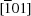	±[101]	± 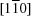	±[110]	± 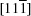	± 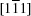	± 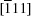	±[111]
**g** = 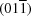	Invisible	Visible	Visible	Visible	Visible	Visible	Visible	Visible	Invisible	Invisible

† The invisibility of these planes is due to their positions either perpendicular or parallel to the specimen surface.
